# Reliability and validity of the Turkish version of the Infant Motor Activity Log in infants with upper extremity functional asymmetry: how often and how well?

**DOI:** 10.55730/1300-0144.5909

**Published:** 2024-10-11

**Authors:** Kübra SEYHAN BIYIK, Cemil ÖZAL, Kıvanç DELİOĞLU, Mintaze KEREM GÜNEL

**Affiliations:** Cerebral Palsy and Pediatric Rehabilitation Unit, Faculty of Physical Therapy and Rehabilitation, Hacettepe University, Ankara, Turkiye

**Keywords:** Asymmetry, brachial plexus, cerebral palsy, infant, upper extremity

## Abstract

**Background/aim:**

Functional asymmetry in the upper extremities may occur in infants with neuromotor problems due to neurodevelopmental or musculoskeletal disorders. The aim of this study was to investigate the validity and reliability of the Turkish version of the Infant Motor Activity Log (IMAL-T), which assesses the frequency (how often) and quality (how well) of the affected arm usage during activities in infants with functional asymmetry in the upper extremities.

**Materials and methods:**

The IMAL-T was administered face-to-face to the parents of 102 infants [60 infants at high risk of developing cerebral palsy (CP) and 42 infants with brachial plexus birth injury (BPBI)], aged 6–24 months, with functional asymmetry in the upper extremities. One week later, the IMAL-T was administered again to 22 parents to determine the test-retest reliability. Cronbach’s alpha and the intraclass correlation coefficient (ICC) were used to determine the internal consistency and test-retest reliability. Discriminant validity was assessed using the manual ability level (Mini Manual Ability Classification System) and the nerve injury type was evaluated using the independent samples t test. For concurrent validity, the relationship between the IMAL-T and the Pediatric Evaluation of Disability Inventory (PEDI) self-care was examined using Spearman’s correlation coefficient.

**Results:**

Internal consistency (Cronbach’s alpha ≥ 0.91) and test-retest reliability (ICC ≥ 0.93) of the IMAL-T were adequate. The IMAL-T scores differed according to the mini-MACS and nerve injury type (p < 0.05). Moderate to strong (CP, r ≥ 0.706, p < 0.001; BPBI, r ≥ 0.579, p < 0.001) correlation coefficients were found between the IMAL-T and PEDI self-care scores.

**Conclusion:**

The IMAL-T is a reliable and valid parent-reported outcome measure that indicates the frequency and quality of the affected arm use during age-appropriate real-life activity in infants aged 6–24 months with upper extremity functional asymmetry due to neuromotor problems. The IMAL-T can be used in early intervention to assess upper extremity functional asymmetry in Turkish infants.

## 1. Introduction

Many self-care activities in daily life require the use of both upper extremities [1]. How often and how well usage of the affected arm affects the quality of bilateral activities [2]. Children with neurodevelopmental problems may present with different clinical pictures depending on their body topography. In cases where only the right or left side of the body is predominantly affected, asymmetry may be noticeable in both posture and function. In neurodevelopmental problems such as cerebral palsy (CP) and brachial plexus birth injury (BPBI), asymmetric involvement is often seen from the earliest years of life [3].

CP is a group of permanent postural and movement disorders associated with nonprogressive impairments in the developing brain that result in activity limitations [4]. As a result of brain lesions, some infants at high risk of developing CP may have markedly increased tone, loss of muscle strength, disuse atrophy, and functional deficits in one side of their body. This clinical picture may develop over time into unilateral CP or asymmetrically affected bilateral CP [5]. Upper extremity functional asymmetric involvement in infants at high risk of developing CP can be identified in the first few months of life with findings such as less looking to one side of the body, less turning, and the inability or reduced ability to grasp objects with the affected hand. Due to developmental reflexes that are not suppressed in infants, the formation of selective and voluntary movements in the affected upper extremity is limited in the first years of life. This begins to interfere with the daily activities at an early age [6,7].

Another group with widespread upper extremity functional asymmetric involvement are infants with BPBI [8]. Asymmetric use of both upper extremities in bimanual activities, developmental delays, or asymmetric development may occur due to nerve injury at birth [9–11]. Depending on the level and severity of the injury, the infant may have difficulty using the affected arm in some daily activities and may not be able to perform some activities at all [12]. With early intervention in BPBI, active/passive joint movement is increased and integration of the affected extremity into the body and functional development are supported [13]. It is very important to monitor the frequency and quality of use of the affected upper extremity in the early stages.

Activities that require upper extremity function, such as playing, feeding, and self-care, are the most intensive areas of participation in infancy [14]. While the use of one hand is sufficient for activities such as grasping a toy and pressing a button on the toy, the use of both upper hands is required for activities such as transferring toys from hand to hand in more complex games, carrying large toys, self-care, and feeding. How often and how well the affected upper extremity participates in these activities in the early years of life will support childhood functional independence [15,16].

Early intervention in the first years of life is valuable for the acquisition of functional skills in both infants at high risk of developing CP and in those with BPBI [17,18]. Scales such as the Melbourne Assessment [19], Quality of Upper Extremity Skills Test [20], and Bayley Scales of Infant and Toddler Development (BSID) [21] are used by experts to demonstrate the efficacy of motor interventions, such as constrained induced movement therapy (CIMT) and intensive bimanual training for functional asymmetry [22]. In addition to expert ratings, parent-reported outcome measures such as the Children’s Hand-use Experience Questionnaire [23], the Hand-Use-at-Home Questionnaire [24], and the Pediatric Motor Activity Log (PMAL) [2] help to assess functional asymmetry in activities of daily living. However, the activities included in these outcome measures are not appropriate for children under two years of age.

As part of CIMT treatment, Taub et al. developed the Motor Activity Logs to examine how often and how well stroke patients and children with CP use their affected arm in their natural environment outside of the clinical setting [2,15,16]. The Infant Motor Activity Log (IMAL), derived from the PMAL, is used globally in infants to assess functional use of the affected upper extremity in age-appropriate real-world activities [25,26]. The IMAL is a unique parent-reported outcome measure that assesses how often and how well the affected upper extremity is used during activities in infants aged 6 to 24 months with upper extremity functional asymmetry [25]. The IMAL is important for physiotherapists to evaluate the effectiveness of upper extremity-focused early interventions in infants with upper extremity involvement [25]. A study conducted by Lowes et al. [26] on the effectiveness of home-based CIMT for infants with CP revealed that the infants showed varying levels of improvement in different items on the IMAL. Therefore, the IMAL items may be important milestones for detailing the functional developmental trajectory of affected arm use in infant’s natural environment and for setting activity goals together with parent/caregiver in infant- and parent-centered early intervention [4,6,7]. On the other hand, there is no Turkish version of this scale or a similar outcome measure that assesses upper extremity functional asymmetry in early intervention management. To use a health-related patient/parent-reported outcome measure in a population with a different language and culture, it is necessary not only to translate it into the target language, but also to demonstrate the validity and reliability of the scale to ensure population-specific standardization of its administration [27]. The aim of this study was to determine the reliability and validity of the Turkish version of the IMAL (IMAL-T) in infants with upper extremity functional asymmetry. The hypotheses were: 1) The IMAL-T is a valid questionnaire for use in Turkish infants with upper extremity functional asymmetry. 2) The IMAL-T is a reliable questionnaire for use in Turkish infants with upper extremity functional asymmetry.

## 2. Materials and methods

### 2.1. Study design

This was a methodological study investigating the reliability and validity of the IMAL-T. The permissions required for the Turkish adaptation, reliability, and validity study of the IMAL-T were obtained from Lowes et al. [26]. This study was conducted at the Cerebral Palsy and Pediatric Rehabilitation Unit, Faculty of Physical Therapy and Rehabilitation, Hacettepe University, between June 2022 and May 2023. Noninterventional clinical research approval was obtained from the Hacettepe University, Nonclinical Research Ethical Board (Number: GO 22/541, Decision No: 2022-09-62). Parents were informed about the study and written informed consent was obtained from those who wished to participate.

### 2.2. Setting and participants

Infants aged 6–24 months who were referred by specialists to the Cerebral Palsy and Pediatric Rehabilitation Unit for physiotherapy and family education and who were reported to have asymmetry in the functional use of the upper extremities, infants at high risk of developing CP or those diagnosed with BPBI, and their parents were included in the study. Infants with additional diagnoses other than a high risk of developing CP or BPBI diagnosis and those with visual and hearing problems, and parents who were illiterate in Turkish, were excluded from the study. Data from parents who were unable to complete the study were excluded during the process.

The total sample size was calculated as recommended by the general guidelines, which require 5–10 individuals for each item to conduct the planned analysis. Furthermore, according to the Consensus-based Standards for the Selection of Health Measurement Instruments (COSMIN) checklist, a sample size of 50–99 is considered good for reliability assessment. At least 20% of the total participants were required for the test-retest [27,28].

One hundred and fifteen parents/caregivers were invited to participate in the study. Of these, 105 parents/caregivers of 61 infants at high risk of developing CP and 44 infants with BPBI agreed to participate in the study. Three parents dropped out of the study due to time constraints. The study was completed with 102 parents/caregivers. Twenty-two of these parents were reinterviewed one week later for test-retest reliability.

### 2.3. Measures

#### 2.3.1. IMAL

The IMAL is a questionnaire designed to ask families how often and how well the affected arm is used during bilateral upper extremity activities in 6–24 month old infants with functionally asymmetric arm involvement. It is a mother/caregiver-reported questionnaire and is administered by an evaluator in a face-to-face interview. It consists of 20 items. Each item is expected to be answered. The 20 items in the questionnaire are selected from bilateral and unilateral upper extremity activities performed by the infants. Scoring ranges from 0; never uses the affected arm to 5; uses the affected arm as well and as often as the other arm. A higher score indicates better quality use of the affected arm, and the maximum score is 100. The validity coefficient (>0.70), internal consistency (Cronbach’s alpha = 0.88) and reliability (0.64–0.70) of the BSID were found to be adequate [25].

#### 2.3.2. Pediatric Evaluation of Disability Inventory (PEDI)

The PEDI is a comprehensive clinical assessment tool that evaluates children’s functional ability and performance by interviewing the family. It is a valid and reliable scale for children aged 6 months to 7.5 years with physical disabilities [29]. The PEDI consists of three main sections: Functional Skills, Caregiver Support, and Modifications. Each of these sections assesses self-care, mobility, and social functioning. Items in each section are scored as 0; cannot and 1; can. At the end of each section, the scores for that section are summed and the total Functional Skills Score is obtained by summing the scores for all the sections. The lowest score is 0 and the highest score is 197. The higher the score, the higher the level of independence. Each of the PEDI subscales can be used independently [30]. The Turkish version of the PEDI was validated and reliable, with a correlation coefficient of 0.86 and an ICC score of 0.96 [31].

#### 2.3.3. Mini Manual Ability Classification System (mini-MACS)

Infants at high risk of developing CP were classified according to the mini-MACS levels. It is a functional classification system that defines bimanual abilities in infants/children with CP on an ordinal scale at 5 levels. Level 1 includes infants/children with independent bimanual functions, while Level 5 includes children who cannot grasp objects and are constantly dependent on another person. The ICC value of the mini-MACS classification is >0.95 [32].

#### 2.3.4. Type of nerve injury

Infants with BPBI were divided into two groups according to the type of nerve injury, as upper trunk and total injury, according to Narakas classification. Narakas classifies BPBI into four groups: Type 1, with C5-C6 spinal nerve injuries; Type 2, with C5-C6-C7 spinal nerve injuries; Type 3, complete paralysis, with C5-T1 spinal nerve injuries; and Type 4, with a combination of C5-T1 spinal nerve injuries and Horner’s syndrome. Narakas Types I and II reflect upper truncal injury and Narakas Types III and IV reflect total injury. Narakas classification is made within the first 2 months of life and does not change thereafter [33].

### 2.4. Procedure

The psychometric properties of the IMAL-T were examined in two phases: 1) the translation phase and 2) the reliability and validity assessment phase. The translation was done by an expert group (two physiotherapists and developers).

#### 2.4.1. Translation and cross-cultural adaptation

Cross-cultural adaptation was conducted according to Beaton’s adaptation guide of cross-cultural adaptation steps [34]. The translation protocol consisted of six steps: forward translation, translation synthesis, back translation, expert committee, focus group, report presentation to developers, and the final version. For forward translation, the scale was translated from English to Turkish by an expert fluent in both languages. The translation was then back-translated from Turkish to English by a bilingual translator and sent to the developers of the IMAL. After the forward- and back-translation were completed, the semantic, idiomatic, empirical, and conceptual equivalence of the scale were discussed and agreed upon by an expert committee (four members) including the developers of the IMAL. This resulted in a preliminary version of the IMAL-T. A focus group of five parents of children with CP and five parents of children with BBPI were asked about the comprehensibility of the items and instructions of the IMAL-T. As there were no items that the parents did not understand or wanted to change, the final version of the IMAL-T was approved after contacting the developers of the original version.

#### 2.4.2. Reliability and validity

Internal consistency, test-retest reliability, discriminant validity (hypothesis testing), and concurrent validity were assessed according to the COSMIN criteria [27]. Physiotherapists (KSB, KD, and CÖ) with at least 10 years of experience in pediatric physiotherapy and rehabilitation recorded the sociodemographic data from the parents and administered the IMAL-T to the parents for reliability and validity. The Narakas type of the infants with BPBI determined in the second postnatal month was obtained from hospital records. Manual ability levels of the infants at high risk of developing CP were assessed according to the mini-MACS by the physiotherapists (KSB and CÖ). In the clinical setting, the parents were informed about the IMAL-T and PEDI, and they were administered in a face-to-face interview. One week later, the IMAL-T was administered again to the same parents by the same investigators under the same settings to examine the presence of temporal stability over time (test-retest reliability).

### 2.5. Data analysis

The data were analyzed using IBM SPSS Statistics for Windows 26.0 (IBM Corp, Armonk, NY, USA). Compliance of the data with normal distribution was assessed visually (probability plots and histograms) and analytically (Kolmogorov–Smirnov/Shapiro–Wilk’s test). Continuous variables were presented as the mean ± standard deviation (SD) or median (min–max). Categorical variables were presented as frequencies and percentages.

#### 2.5.1. Internal consistency

The internal consistency of the IMAL-T was examined using Cronbach’s alpha (α) values: α ≥ 0.90 indicates excellent consistency, 0.80 ≤ α < 0.90 indicates good consistency, and 0.70 ≤ α < 0.80 indicates acceptable consistency. The acceptable value for Cronbach’s alpha is 0.70 [27].

#### 2.5.2. Test-retest reliability

The intraclass correlation coefficient (ICC) was used to measure test-retest reliability. The ICC values were defined as follows: <0.50, poor; between 0.50 and 0.75, moderate; between 0.75 and 0.90, good; and >0.90 excellent reliability [35]. Adequate test-retest reliability does not necessarily ensure that the participants’ repeated answers will be consistent from test to test. Therefore, the minimal detectable change (MDC) and the standard error of measurement (SEM) were calculated to provide absolute reliability. The SEM provided a measure of variability but was primarily used for calculating the MDC. The SEM values were calculated as follows: SEM = SD × √(1 – ICC), MDC of each tool with the following formula: MDC = z-score [95% confidence interval (CI)] × SEM × √2 [36].

#### 2.5.3. Concurrent validity

Spearman’s rank correlation was used to determine the association between baseline IMAL-T and PEDI self-care. A Spearman’s rank correlation coefficient ≥0.80 was defined as very strong, 0.80 to 0.60 as strong, 0.60 to 0.40 as moderate, 0.40 to 0.20 as weak, and <0.20 as very weak [37].

#### 2.5.4. Discriminant validity

It was conducted to evaluate whether the IMAL-T has discriminatory ability in known groups [27]. Infants at high risk of developing CP were divided according to the mini-MACS levels and infants with BPBI were divided into upper trunk and total injury groups according to the type of nerve injury. Changes between the groups were analyzed using the independent samples t test. Statistical significance was accepted as p < 0.05.

## 3. Results

Of the 102 infants with unilateral upper extremity involvement, 60 (58.8%) had a high risk of developing CP and 42 (41.2%) had a BPBI diagnosis. The mean age range was 12.34 ± 6.79 years (6–24 months) and more than half of the infants were male (52%). The mean age of the parents was 26.08 ± 5.71 (18–36) years. Most of the parents were high school or university graduates (Table 1).

### 3.1. Internal consistency and test-retest reliability

The internal consistency of the IMAL-T How Often and How Well scales was adequate (Cronbach’s alpha > 0.70). Table 2 demonstrates the ICC, 95% CI, SEM, and MDC. The test-retest reliability of the IMAL-T How Often and How Well scales was high (Table 2).

### 3.2. Concurrent validity

A positive moderate to strong relationship was found between the IMAL-T How Often and How Well scales and the PEDI self-care scores (p < 0.001, Table 3).

### 3.3. Discriminant validity

**Figure 1:**
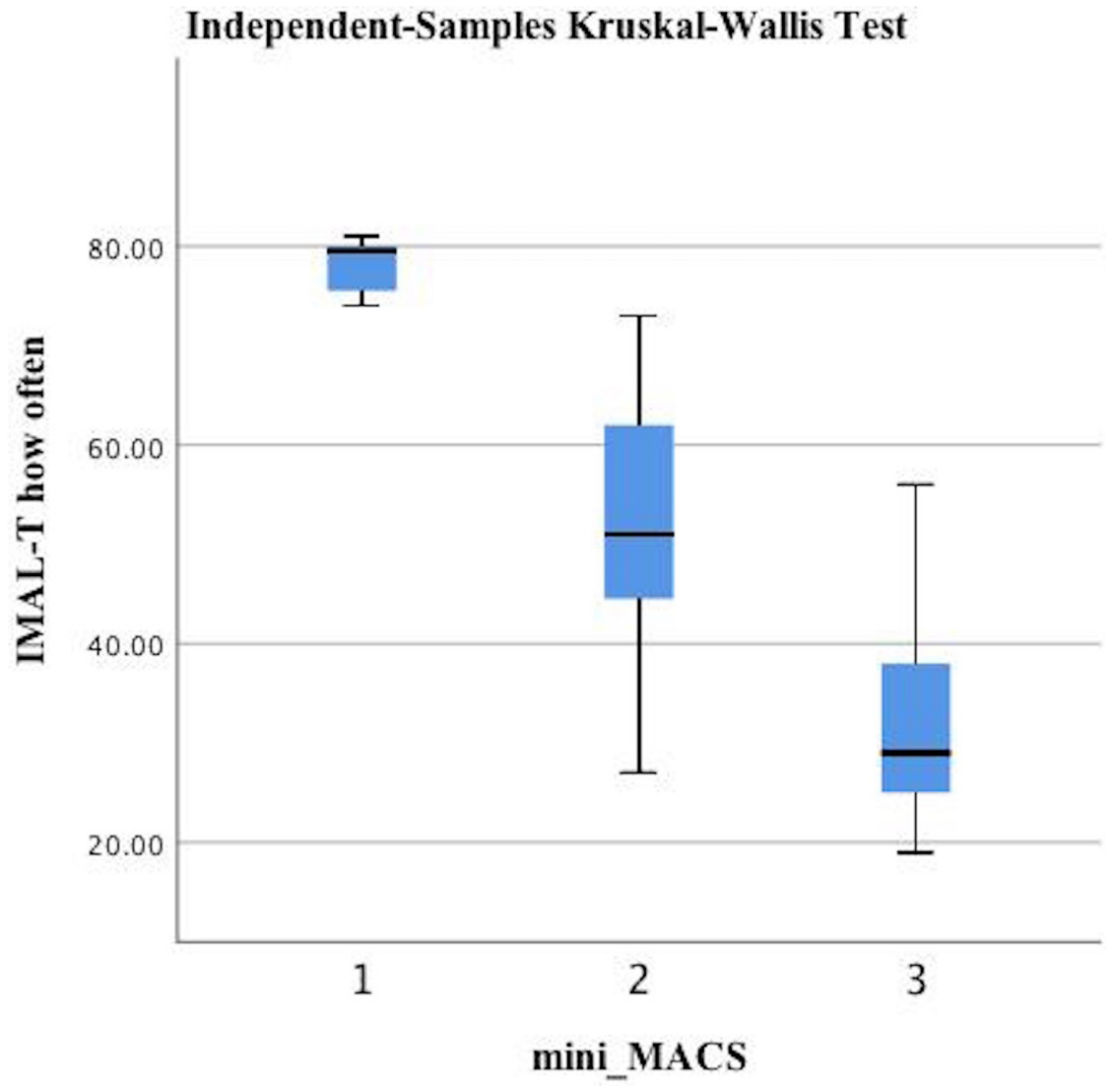
Comparisons of the IMAL-T How Often score according to the mini-MACS levels.

**Figure 2:**
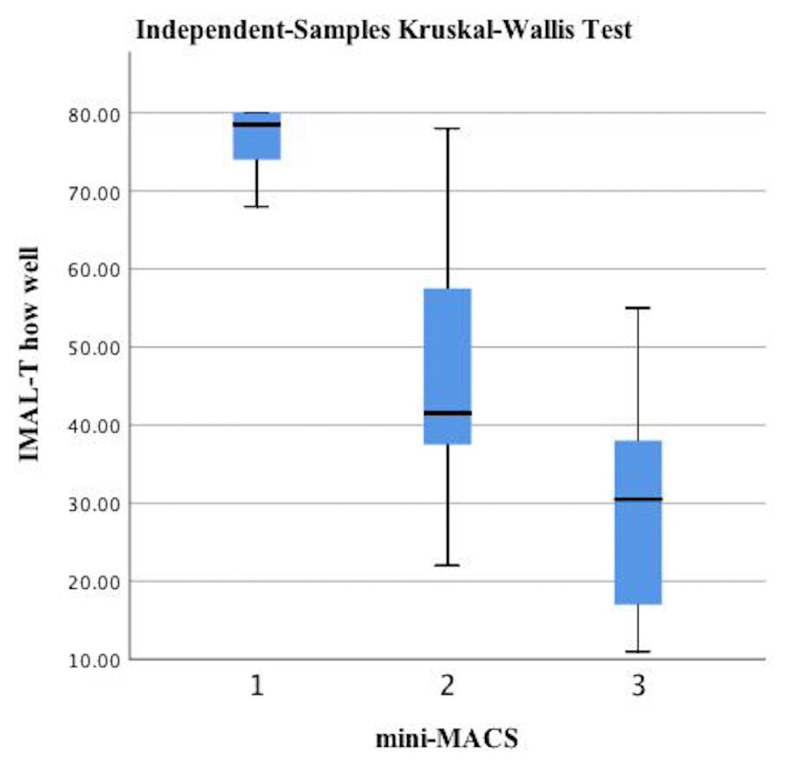
Comparisons of the IMAL-T How Well score according to the mini-MACS levels.

**Figure 3:**
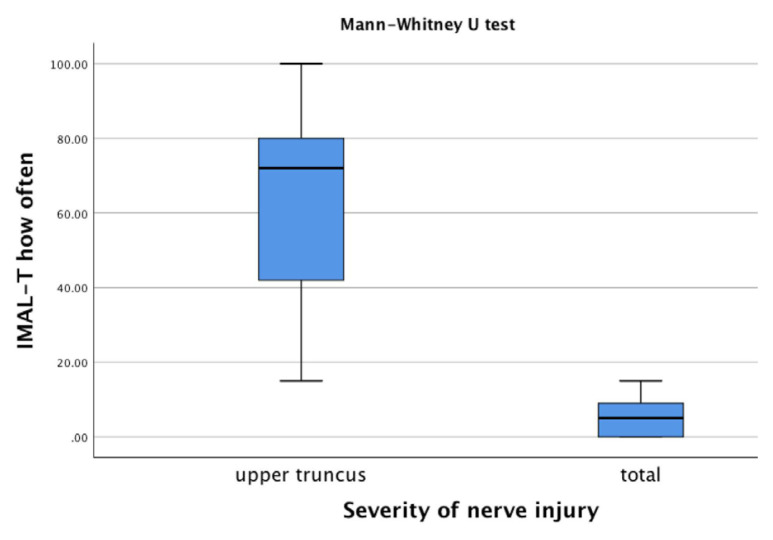
Comparisons of the IMAL-T How Often score according to the severity of nerve injury.

**Figure 4:**
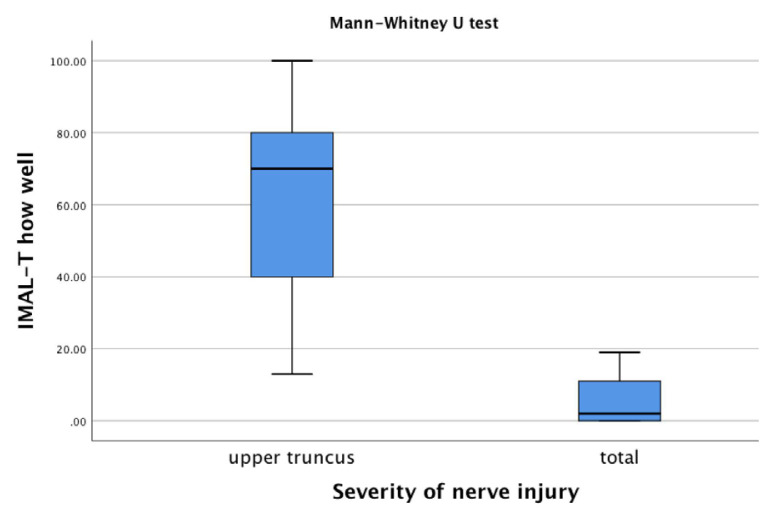
Comparisons of the IMAL-T How Well score according to the severity of nerve injury.

According to the mini-MACS, infants at high risk of developing CP were classified between levels I and III. The IMAL-T How Often and How Well scores decreased from level I to level III based on manual ability (p < 0.05, Figures 1 and 2, and Appendix). The difference between the upper truncus and total injury groups in the infants with BPBI was also statistically significant (p < 0.05). The upper truncus group had higher IMAL-T How Often and How Well scores than the total injury group (Figures 3 and 4, and Appendix).

## 4. Discussion

The Turkish version of the IMAL, the unique parent-reported outcome measure that assesses the frequency (how often) and quality (how well) of affected arm functional usage in infants aged 6–24 months with upper extremity functional asymmetry due to neurodevelopmental disabilities, had excellent internal consistency and test-retest reliability. Moderate to very strong correlation coefficients were found between the IMAL-T subscores and PEDI self-care score. The IMAL-T was able to discriminate infants at high risk of developing CP according to the mini-MACS levels and infants with BPBI according to the severity of nerve injury.

Internal consistency was defined as the relationship between items in a scale [27]. The IMAL is a revised version of the items in the PMAL, which is used to assess functional asymmetry of the upper extremities in children older than two years of age. The internal consistency of the PMAL, as used in children older than two years of age, was found to be adequate in both children with CP and those with BPBI in terms of the How Often and How Well scales [38,39]. Carey et al. [25] reported a Cronbach’s alpha of ≥0.88 for both the How Often and How Well scales in the internal consistency of the IMAL in infants with functional impairment and those at risk of developing CP. In the current study, the internal consistency of the Turkish version of the PMAL-inspired IMAL was adequate (Cronbach’s alpha ≥ 0.91) in infants aged 6–24 months with BPBI and those at high risk of developing CP.

The test-retest consistency indicated that the scale is temporally (over time) stable in self-reported outcome measures [40]. Test-retest reliability of the PMAL was high in children with CP and in those older than two years of age with BPBI [38,39]. Test-retest reliability of the original version of the IMAL was also adequate in infants with functional impairment [25]. In the current study, test-retest reliability was high in infants younger than two years of age with BPBI and in those at high risk of developing CP. The scores of the IMAL-T were shown to have sufficient stability over time. The SEM value expresses the acceptable difference between the expected and measured values in a measurement [36,41], so when using the IMAL in infants with upper extremity functional asymmetry, a change of 3 points in the How Well scale and a change of 6 points in the How Often scale can be considered as an error of measurement. The MDC value represents the measure of statistically significant change between two independently obtained measurements, so if an 8-point difference in the How Well scale and a 19-point difference in the How Often scale occurs between two assessments during medical process follow-up, this represents a statistically significant change.

Discriminant validity using hypothesis testing is analyzed by comparing participants in a known group (subgroup) analysis. Meaningful changes are seen between relevant subgroups (e.g., patients with expected high versus low levels of the construct of interest) [42]. MACS levels are used for discriminative validity in methodological studies related to upper extremity functionality in children with CP [43]. Carey et al. [25] reported that the IMAL scores decreased as the mini-MACS levels increased, supporting discriminant validity in their study. In the present study, the IMAL-T scores differed significantly according to the mini-MACS levels. The IMAL-T scores were higher at lower mini-MACS levels. The IMAL-T had discriminant validity according to the manual ability levels of infants at high risk of developing CP. In children with BPBI, the type of nerve injury has been used to demonstrate discriminant validity in upper extremity functional scales [41]. In the present study, the IMAL-T scores of the upper trunk injury group were significantly higher than those of the total nerve injury group. The IMAL-T had discriminant validity according to the type of nerve injury in the infants with BPBI.

Self-care includes activities that require functional use of both hands in daily living, such as eating food and dressing, and also provides information about manual skill levels. Carey et al. [25] found the correlation of the IMAL How Often and How Well scales with the BSID, Third Edition to be 0.70 and 0.72, respectively. Günel et al. [39] used the PEDI for concurrent validity of the PMAL. The PEDI is a parent-reported outcome measure similar to the IMAL in regard to the type of administration. It can be used in infants with both CP and BPBI after 6 months of age [30]. There is a strong positive relationship between the PEDI self-care subscale and bimanual performance in children with CP [44]. In the present study, the self-care subscale of the PEDI was used for concurrent validity, given the characteristics of the population and the scales. As the IMAL-T How Often and How Well scores increased, the PEDI self-care scores also increased. Moderate to strong correlation coefficient values were obtained between the IMAL-T and the PEDI self-care scale in infants at high risk of developing CP and those with BPBI.

This study had several limitations. The major limitation was that it did not include typically developing infants. In addition, infants at high risk of developing CP had only mild to moderate manual ability involvement (mini-MACS levels I-III), limiting the generalizability of the discriminative characteristics of the IMAL-T to all manual ability levels. Furthermore, manual classification of infants less than one year of age was not available in the literature, resulting in the exclusion of infants less than one year of age from the discriminant validity. Future studies are recommended to investigate the validity and reliability of the IMAL/IMAL-T in infants with upper extremity functional asymmetry with different diagnoses or congenital malformations. It is also recommended to include typically developing infants to discriminate the difference in real-life functional arm use between infants with and without upper extremity involvement. Further methodological studies on infants at risk of developing CP should also include infants at MACS levels IV and V. There is also a need for studies investigating other methodological properties (responsiveness, etc.) of the IMAL-T scale.

The IMAL-T is a reliable and valid parent-reported outcome measure for assessing infants aged 6–24 months with upper extremity functional asymmetry due to neuromotor problems. The IMAL-T can be used as an early intervention to assess how often and how well the affected extremity is used during unilateral/bilateral functional upper extremity activities in Turkish infants at high risk of developing CP or infants with BPBI.

## Figures and Tables

**Table 1:** Characteristics of the participants.

	Mean ± SD	Range
Age of the infants (months)	12.34 ± 5.79	6–24
Age of the parents (years)	26.08 ± 5.71	18–36
	Frequency	%
Infants with neuromotor problems		
Infants at high risk of developing CP	60	58.8
Infants with BPBI	42	41.2
Involvement side		
Right	62	60.8
Left	40	39.2
Sex of the infants		
Girl	49	48
Boy	53	52
Education level of the parents		
Primary school	2	2
Elementary school	5	4.9
High school and junior college	60	58.8
University	35	34.3

CP, cerebral palsy; BPBI, brachial plexus birth injury

**Table 2:** Internal consistency and test-retest reliability results.

	Internal consistency	Test-retest reliability	95% CI
	Cronbach’s alpha	ICC	Lower–upper bounds
All infants			
IMAL-T How Well	0.954	0.986	0.970–0.994
IMAL-T How Often	0.936	0.950	0.890–0.977
Infants at high risk of developing CP			
IMAL-T How Well	0.939	0.981	0.955–0.993
IMAL-T How Often	0.911	0.935	0.843–0.974
Infants with BPBI			
IMAL-T How Well	0.975	0.998	0.985–0.999
IMAL-T How Often	0.971	0.994	0.963–0.997

IMAL-T, Turkish version of the Infant Motor Activity Log

**Table 3:** Relationships among the IMAL-T How Often and How Well scores, and the PEDI self-care score.

		All infants		Infants at high risk of developing CP n = 60		Infants with BPBI N = 42	
	IMAL-T	How Often	How Well	How Often	How Well	How Often	How Well
PEDI-SC	r	0.683*	0.675*	0.709*	0.706*	0.616*	0.579*
	p-value	<0.001	<0.001	<0.001	<0.001	<0.001	<0.001

* Spearman’s correlation coefficient;

PEDI-SC; Pediatric Evaluation of Disability Inventory self-care.

**Appendix:** Comparisons of the IMAL-T How Often and How Well scores according to the mini-MACS (infants at risk for CP) and type of nerve injury (infants with BPBI).

Infants at high risk of developing CP, n = 42							
IMAL-T How Often				IMAL-T How Well			
Level I (n = 16)	Level II (n = 12)	Level III (n = 14)		Level I (n = 16)	Level II (n = 12)	Level III (n = 14)	
Median (min–max)	Median (min–max)	Median (min–max)	*p-value	Median (min–max)	Median (min–max)	Median (min–max)	*p-value
79.5 (74–81)	51 (27–73)	26 (19–38)	<0.001	78.5 (68–80)	41.5 (22–68)	23 (11–55)	<0.001
Infants with BPBI, n = 42							
IMAL-T How Often				IMAL-T How Well			
Upper truncus injury (n = 33)	Total nerve injury (n = 9)			Upper truncus injury (n = 33)	Total nerve injury (n = 9)		
Median (min–max)	Median (min–max)		**p-value	Median (min–max)	Median (min–max)		**p-value
72 (15–100)	5 (0–15)		<0.001	70 (13–100)	2 (0–19)		<0.001

* Kruskal–Wallis test, pairwise comparisons were significant between levels I–II, II–III, and I–III;

** Mann–Whitney U test.
